# Natural monoterpenoid geraniol promotes antioxidant defense and stress tolerance via SKN-1/Nrf2 activation in *Caenorhabditis elegans*

**DOI:** 10.1007/s13659-026-00604-4

**Published:** 2026-04-22

**Authors:** Stéfano Romussi, Ailin Lacour, Diego Rayes, María José De Rosa

**Affiliations:** 1 Laboratorio Neurobiología de Invertebrados, Instituto de Investigaciones Bioquímicas de Bahía Blanca (INIBIBB) CCT UNS-CONICET, 8000, Camino La Carrindanga Km 7, Buenos Aires, 8000, Bahía Blanca, Argentina; 2 Departamento de Biología, Bioquímica y Farmacia, Universidad Nacional del Sur (UNS), 8000, San Juan 670, Buenos Aires, 8000, Bahía Blanca, Argentina

**Keywords:** *Caenorhabditis elegans*, Antioxidant, Essential oil, Cytoprotective mechanism, Oxidative stress

## Abstract

Oxidative stress (OS) is a major contributor to aging and the pathogenesis of numerous conditions, including diabetes, neurodegenerative, cardiovascular, and autoimmune disorders. Consequently, therapeutic strategies aimed at enhancing endogenous cytoprotective pathways have gained significant interest. Plant-derived essential oils represent attractive sources for such interventions due to their natural origin and low toxicity; specifically, the monoterpenoid geraniol, a principal component of rose oil, has demonstrated promising antioxidant properties in vitro. In this study, we utilized *Caenorhabditis elegans* to investigate the in vivo efficacy and molecular mechanisms of geraniol effect. Our results show that geraniol significantly reduces intracellular reactive oxygen species and enhances resistance to acute OS induced by juglone. Mechanistic characterization using GFP-reporter strains revealed that geraniol activates the DAF-16/FOXO and SKN-1/Nrf2 transcription factors, while surprisingly causing a slight but consistent downregulation of the HSF-1-mediated heat-shock response. Crucially, genetic epistasis analysis using null/hypomorphic mutants demonstrated that only SKN-1 is strictly essential for geraniol-mediated protection against induced OS. In conclusion, this study underscores the utility of *C. elegans* as a robust and accessible platform for the pharmacological screening of natural products. Our findings establish geraniol, a key constituent of rose oil, as a multifunctional modulator of cellular defenses that orchestrates multiple cytoprotective pathways, identifying the SKN-1-dependent response as a critical driver of its antioxidant efficacy in *C. elegans*. By delineating this specific genetic requirement, these results provide a mechanistic foundation that supports the therapeutic potential of geraniol in mitigating aging and pathophysiology driven by OS.

**Graphical abstract:**

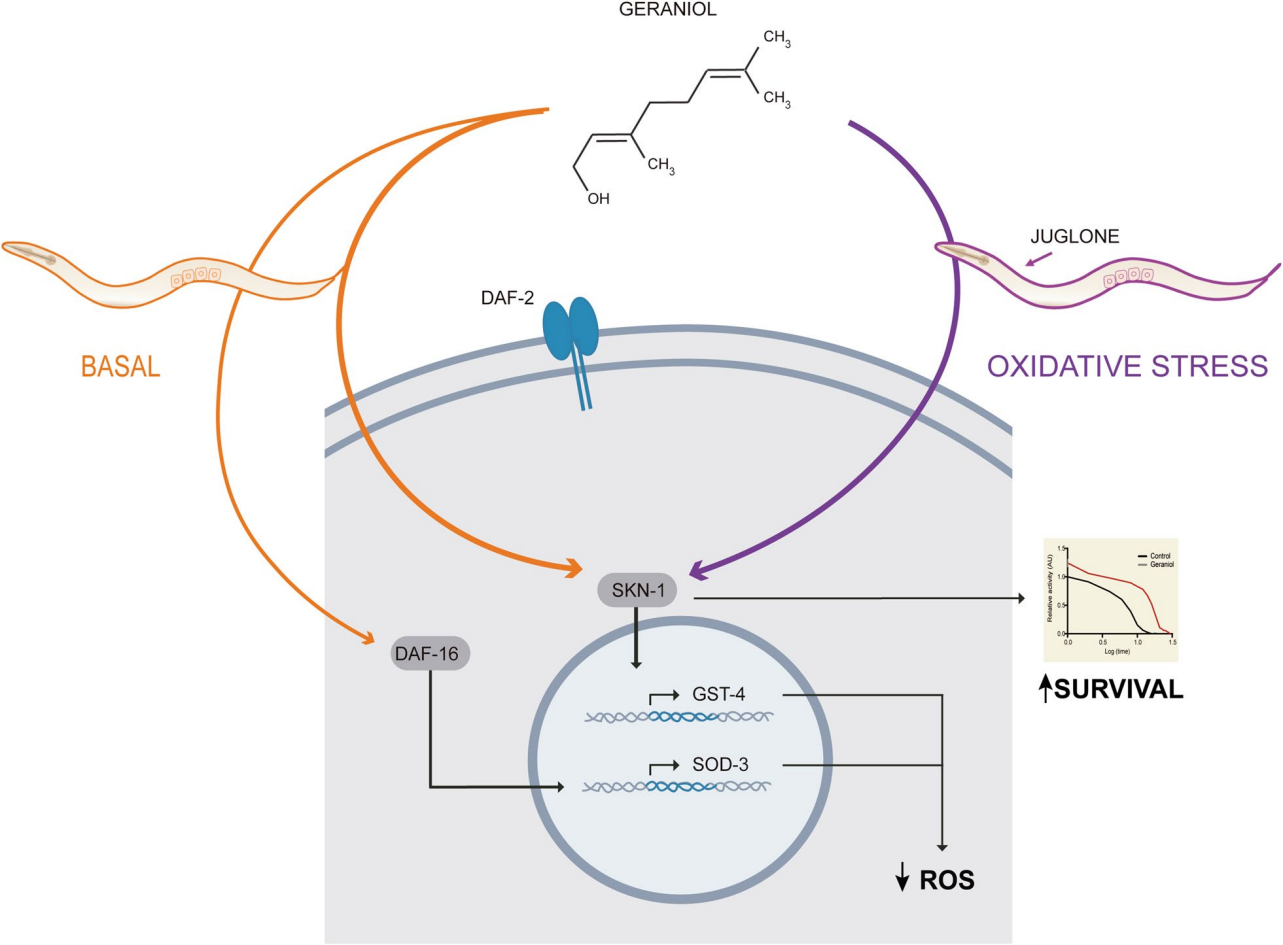

**Supplementary Information:**

The online version contains supplementary material available at 10.1007/s13659-026-00604-4.

## Introduction

Oxidative stress (OS), arising from an imbalance in redox homeostasis, is a major contributing factor in aging and the pathogenesis of a broad spectrum of diseases ranging from neurodegenerative disorders (such as Alzheimer's and Parkinson's diseases) and cardiovascular ailments to diabetes and autoimmune pathologies like rheumatoid arthritis [1–3]. OS is characterized by the excessive accumulation of reactive oxygen species (ROS), which inflict damage on cellular components and drive chronic inflammation [4, 5]. To deal with ROS accumulation, organisms activate endogenous cytoprotective pathways designed to neutralize them and mitigate cellular injury. Therefore, a central therapeutic strategy for modulating aging and disease risk involves enhancing these antioxidant defenses—either by directly inactivating ROS through scavenger molecules or by pharmacologically inducing these intrinsic protective mechanisms [6].

Plant-derived essential oils are natural compounds extracted from various parts of plants, including flowers, leaves, stems, and roots. These oils are typically composed of a diverse array of molecules, including terpenoids, phenylpropenes, terpenes, ketones, aldehydes, esters, and phenols [7]. Many of them have gained recognition for their antioxidant properties [8]. Herbal compounds, such as those found in essential oils, are increasingly being explored as potential alternatives to synthetic drugs, owing to their widespread consumption through dietary inclusion and their relatively low toxicity. Despite their potential, the complexity of plant essential oils has made it challenging to isolate specific bioactive components, hindering a full understanding of their molecular mechanisms and delaying their clinical application.

Geraniol, an acyclic monoterpenoid and a principal component of essential oils from plants such as rose, lemon, and ginger [9], presents a compelling candidate for therapeutic investigation. Beyond its widespread use as a fragrance in cosmetics and household products, geraniol is characterized by low toxicity and favorable bioavailability [10]. Notably, both in vitro and in vivo studies have demonstrated promising pharmacological properties, including antioxidant, cytoprotective, and anticancer effects [10, 11]. These findings underscore its potential as a bioactive molecule for intervention in diseases where such mechanisms are relevant. Despite this potential, the specific cellular and molecular pathways through which geraniol exerts its cytoprotective effects have yet to be fully elucidated.

The nematode *Caenorhabditis elegans* has become a powerful model organism in biomedical research, owing to its short lifespan, genetic tractability, and anatomical simplicity, which collectively facilitate the study of fundamental biological processes and disease mechanisms [12, 13]. Its transparency allows for real-time visualization of cellular and molecular events using fluorescent reporters and biosensors [14, 15]. Notably, research in *C. elegans* has been instrumental in identifying conserved genetic pathways that regulate OS resistance, autophagy, and longevity [16, 17]. A cornerstone finding in this model is the DAF-2/insulin/IGF-1 signaling (IIS) pathway, a universal key regulator of lifespan and proteostasis [18]. The IIS pathway converges on key cytoprotective transcription factors, including DAF-16, the *C. elegans* ortholog of mammalian FOXO; SKN-1, the ortholog of mammalian Nrf1/Nrf2; and HSF-1, the ortholog of mammalian HSF. Their activation can ameliorate phenotypic deficits in various *C. elegans* disease models [12, 19–24]. Consequently, *C. elegans* has become a powerful platform for screening and characterizing genes, drug targets, and compounds with therapeutic potential [25, 26].

In this study, we employed the animal model *C. elegans* model to investigate the in vivo antioxidant capacity of the plant-derived compound geraniol and elucidate its underlying molecular mechanisms. Using genetic and transgenic approaches, we demonstrate that geraniol significantly reduces intracellular ROS and enhances resistance to induced OS. We found that exposure to geraniol activates two key cytoprotective transcription factors, DAF-16/FOXO and SKN-1/Nrf2. In contrast, it slightly inhibits the activity of the HSF-1 transcription factor. Interestingly, genetic analysis using null/hypomorphic mutants reveals that the antioxidant effect is dependent specifically on SKN-1, indicating that this pathway is key for geraniol-mediated resistance. Collectively, our findings establish geraniol as a potent activator of endogenous antioxidant defenses in vivo, acting primarily through the SKN-1/Nrf2 axis, and highlight its potential as a therapeutic candidate for diseases driven by OS.

## Results

### Geraniol reduces oxidative stress under basal conditions and upon exogenous challenge

**Fig. 1:**
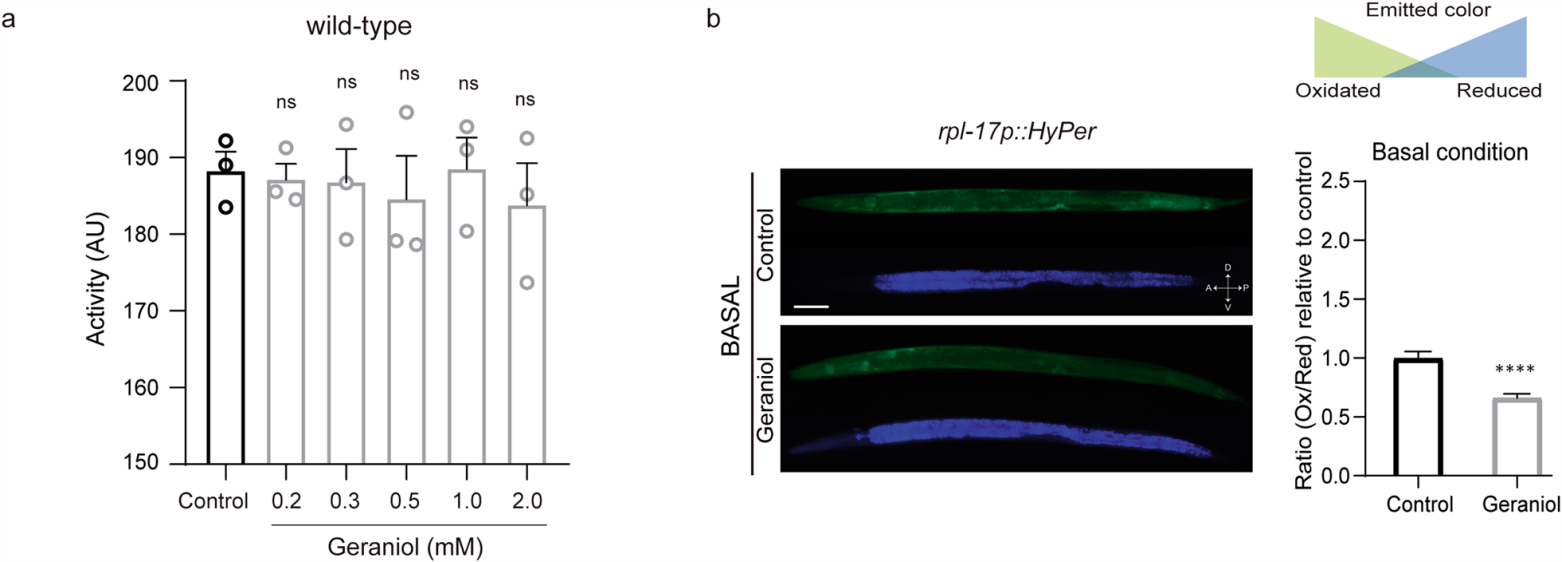
Effect of geraniol in wild-type *C. elegans.* **a** Locomotion of adult day 1 wild-type animals in the absence of geraniol (control, bars with black lineouts) or in the presence of different geraniol concentrations (0.2, 0.3, 0.5, 1.0 and 2.0 mM, bars with grey lineouts), assessed using the WMicrotracker. The graph shows locomotion (in Arbitrary Units (AU)) measured at minute 165 (15-min acquisition interval). The experiment was performed in 3 independent replicates, each including 6–8 wells (approximately 50 worms per well). Statistical significance was determined using one-way ANOVA followed by Holm–Sidak’s post hoc test for multiple comparisons against the control group, and no significant differences (ns) were detected. **b** ROS levels were measured in L4-stage animals previously exposed to DMSO (control condition) or 1 mM geraniol. Left: In vivo microphotographs of JV1 (Hyper) animals using excitation filters at 488 nm (green emission) and 400 nm (blue emission). Right: Intracellular ROS production is expressed as the oxidized/reduced HyPer ratio (green/blue) relative to the control condition. The experiment was performed in 3 independent replicates, each including 10–12 worms. Statistical significance was assessed using the Mann–Whitney test (****) *p* < 0.0001. Data are presented as mean ± SEM

Given that several essential oils have demonstrated anthelmintic properties, and because *Caenorhabditis elegans* is a free-living, non-parasitic nematode, we first performed a dose–response assay (0.2–2 mM) to identify non-toxic concentrations of geraniol for subsequent experiments. To measure potential toxicity, we quantified locomotor activity at a single time point (165 min) using a WMicrotracker, which allows automated quantification of activity in large populations [27]. Continuous exposure from eggs to adult day 1 (AD1) to geraniol concentrations up to 2 mM did not significantly alter locomotor activity, indicating an absence of toxicity or locomotor stimulation at these doses (Fig. 1a). Based on these results, we selected 1 mM as an intermediate working concentration for further analyses.

To assess the potential antioxidant properties of geraniol, we first measured whether chronic exposure to the compound, in the absence of any external stressor, could alter the baseline ROS levels in vivo. Real-time measurements of H_2_O_2_ were obtained using the genetically encoded HyPer sensor, expressed in established transgenic reporter strains. HyPer is a highly specific H_2_O_2_ biosensor whose fluorescence properties change upon oxidation [28, 29]. We measured fluorescence using excitation filters of 488 nm for the oxidized state and 400 nm for the reduced state.

As shown in Fig. 1b, animals treated with geraniol from eggs to the L4 stage exhibited a significant reduction in basal ROS levels, reaching around 66% of those of control animals (66.09% ± 19.54 compared to control animals). This result demonstrates that geraniol possesses intrinsic antioxidant activity in vivo under standard physiological conditions.

**Fig. 2:**
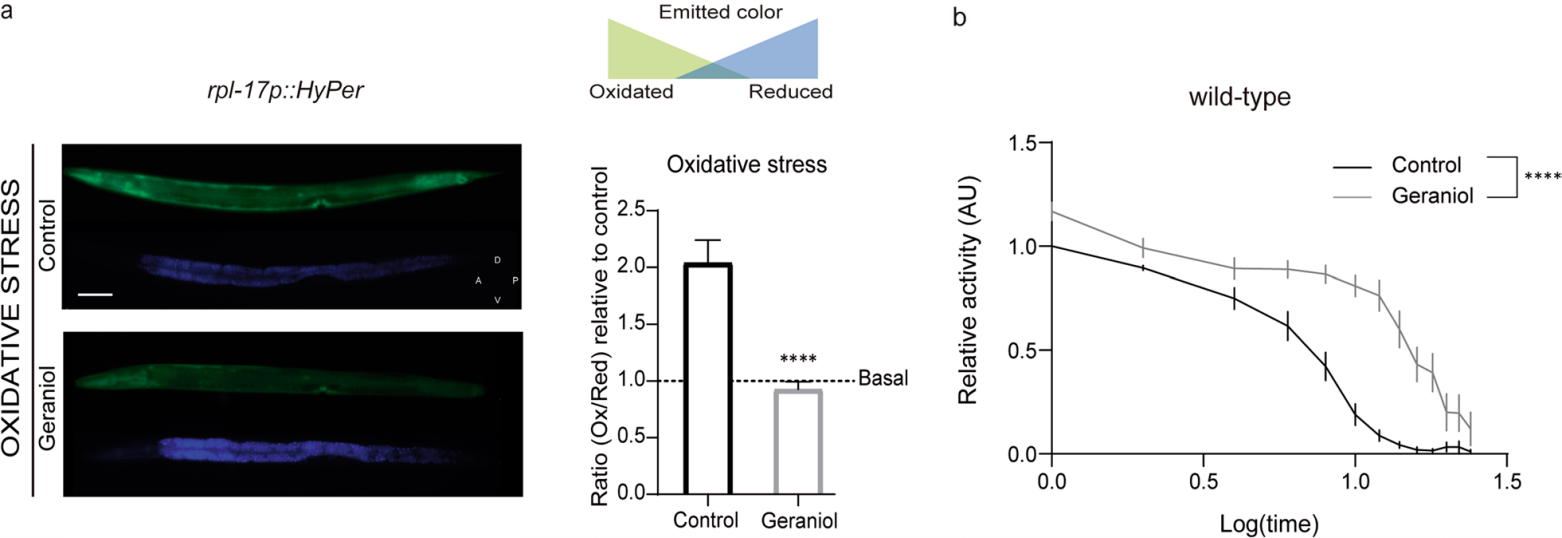
Geraniol confers protection against oxidative stress. **a** ROS levels were measured in L4-stage animals previously exposed to DMSO (control condition) or 1 mM geraniol. Left: In vivo microphotographs of JV1 (Hyper) animals under mild xenobiotic stress (juglone 200 µM, 20 min). Right: Intracellular ROS production is expressed as the oxidized/reduced HyPer ratio (green/blue) relative to control under basal condition. The dashed line indicates the mean of the control under basal conditions. The experiment was performed in 3 independent replicates, each including 10–12 worms. Statistical significance was assessed using the Mann–Whitney test (****) *p* < 0.0001. Data are presented as mean ± SEM. **b** Animal locomotion in the presence of the oxidizing agent juglone (240 μM). The experiments were performed on aged-synchronized animals (adult day 1 stage) exposed to 1 mM geraniol (grey line) or DMSO (black line) throughout development. The experiment was repeated at least 3 independent times using 8–12 wells (approximately 50 worms per well). Data are presented as mean locomotor activity normalized to the first hour of the control condition ± SEM. T50 values were compared using a *t*-test (****) *p* < 0,0001

To further assess geraniol’s protective effects under exogenous oxidative challenge, we used two experimental approaches. First, using transgenic HyPer animals, we monitored ROS levels after a 20 min exposure to juglone (200 µM) in animals pre-treated with either 1 mM geraniol or vehicle (DMSO) (Fig. 2a). Second, using wild-type animals, we assessed locomotor activity as a proxy for survival over a 20h period following the same pre-treatment and juglone challenge (Fig. 2b).

Juglone induced a marked increase in ROS in untreated animals (around 90% above baseline). In contrast, geraniol-treated animals showed a significantly smaller increase (only around 40% above their own baseline) (Additional file 1: Fig. S1 and S2a). Moreover, while juglone-treated animals gradually lost activity and ultimately died, geraniol-pretreated animals displayed substantially improved survival. The time to reach 50% activity loss (T50) was 7.06 h (95%CI 6,48–7,63) for untreated animals and 15.30 h (95%CI 13,88–16,59) for geraniol-treated animals (Fig. 2b).

Together, these findings indicate that geraniol not only lowers basal OS but also confers significant protection against severe, chemically induced oxidative damage.

### Geraniol robustly activates SKN‑1/Nrf2 and enhances DAF‑16/FOXO stress signaling

**Fig. 3:**
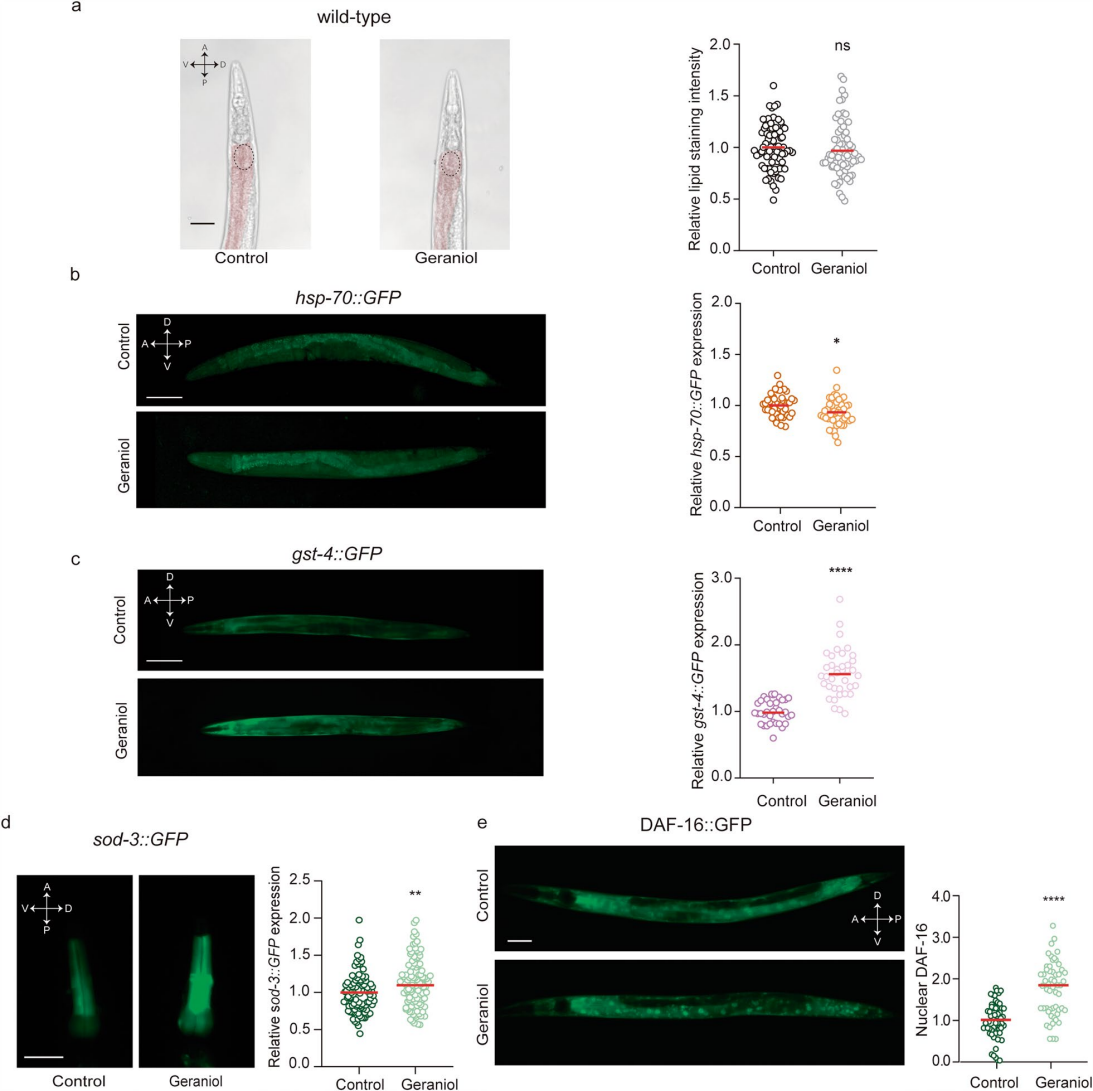
Geraniol activates multiple cytoprotective mechanisms.** a** Left: Representative images of adult day 1 control and geraniol-treated animals after Oil-Red O staining. The circled ROIs indicate the areas in which lipid staining intensity was evaluated. Scale bar, 25 μm. Right: Quantification of relative lipid staining intensity. Black open circles: DMSO-treated animals and grey open circles: geraniol-treated animals. **b** Left: Expression of *hsp-70* was determined using the BC10060 *dpy-5(e907) I; sEx884 [rCesC12C8.1::GFP* + *pCeh361]* strain. Representative images of control (DMSO) and geraniol-treated animals. Scale bar, 50 μm. Right: Relative *hsp-70::GFP* fluorescence quantification in adult day 1 animals. Brown open circles: DMSO-treated animals and orange open circles: geraniol-treated animals. **c** Left: Representative images of adult day 1 control (DMSO) and geraniol-treated animals using CL2166 *dvIs19 [(pAF15)gst-4p::GFP::NLS]* III strain. Scale bar, 100 μm. Right: Quantification of *gst-4:: GFP* expression in adult day 1 animals relative to the control condition. Purple open circles: DMSO-treated animals and pink open circles: geraniol-treated animals. **d** Left: Representative images of the head region of CF1553 *muIs84 [(pAD76) sod-3p::GFP* + *rol-6].* Scale bar, 25 μm. Right: Quantification of *sod-3::GFP* expression in adult day 1 animals in the pharyngeal region, expressed relative to the control condition. **e** Left: Representative images of adult day 1 animals control (DMSO) or geraniol-treated animals using TJ356 *zIs356 [daf-16p::daf-16a/b::GFP* + *rol-6(su1006)]* strain. Scale bar, 50 μm. Right: Nuclear DAF-16 quantification relative to control condition. For **d** and **e**: Dark green open circles: DMSO-treated animals and light green open circles: geraniol-treated animals. For **a**–**e** The experiment was performed in at least 3 independent replicates, each including 10–12 worms. Quantification graphs show the mean value. Statistical significance was assessed using the Mann–Whitney test, (ns: no significant differences, *p* > 0.05 ; (*) *p* < 0.05 ; (**) *p* < 0.01 ; (****) *p* < 0.0001)

Given that geraniol modulates OS in vivo, we used *C. elegans* to investigate the underlying molecular mechanisms. Dietary restriction is known to increase stress resistance and lifespan, and typically manifests as reduced lipid accumulation [17, 30, 31]. We therefore wondered whether geraniol might elicit a dietary restriction-like effect. Oil-Red O lipid staining of AD1-stage worms, however, revealed no significant differences in lipid content between geraniol-treated and control animals, ruling out dietary restriction as the primary mechanism of geraniol’s protective effects (Fig. 3a).

Next, we explored plausible intracellular mechanisms that could explain geraniol-mediated OS protection. To this end, we focused on the cytoprotective activity of transcription factors (TFs) that act downstream of the highly conserved DAF-2/IIS such as DAF-16/FOXO, SKN-1/Nrf-2, and HSF-1/HSF1 [32]. These TFs activate a genetic program that ultimately modulate scavenging mechanisms for free radicals and oxidative metabolites and confer enhanced stress resistance and longevity [12, 33]

We further examined the activation of these key cytoprotective TFs, using transgenic reporter strains to assess the expression of their canonical downstream targets.

We first evaluated HSF‑1 activity by measuring the expression of *hsp‑70*, a canonical transcriptional target and well-established effector of this pathway [34]. Surprisingly, geraniol treatment resulted in a moderate but significant downregulation of *hsp‑70* expression under basal conditions (Fig. 3b). This indicates that geraniol does not activate the HSF‑1 pathway and may, in fact, exert a mild suppressive effect on its basal activity.

In contrast, geraniol exposure strongly induced the expression of glutathione S‑transferase 4 (GST‑4), a direct transcriptional target of SKN‑1, nearly doubling its expression compared to control animals. This result demonstrates that geraniol robustly activates the SKN‑1/Nrf2 pathway under basal conditions (Fig. 3c).

To assess DAF‑16 activity, we first examined the expression of its target gene, superoxide dismutase 3 (SOD‑3). Under basal conditions, geraniol induced a modest but detectable increase in SOD‑3 expression, suggesting DAF‑16 activation (Fig. 3d). To further confirm this, we used a strain expressing a translational reporter (*Pdaf‑16::DAF‑16::GFP*) to visualize DAF‑16 nuclear translocation, a hallmark of its activation. Although nuclear localization of DAF‑16 is generally low under basal conditions (consistent with previous reports [35, 36]), we observed enhanced activation of DAF‑16 following a mild oxidative stimulus (juglone 200 μM for 10 min). Geraniol-treated animals exhibited a significantly greater increase in DAF‑16 nuclear translocation compared to controls (Fig. 3e). This indicates that geraniol not only activates DAF‑16 under basal conditions (as shown by increased SOD‑3 expression), but also potentiates its nuclear translocation in response to OS.

Together, these results establish geraniol as a multimodal modulator that differentially tunes cellular defense programs: it drives constitutive activation of SKN-1/Nrf2, exerts a context-dependent modulation of DAF-16/FOXO activity that is amplified under OS, and subtly attenuates the HSF-1/HSP-70 pathway.

### SKN-1/Nrf2 is essential for geraniol-mediated oxidative stress resistance

To determine which of the transcription factors modulated by geraniol are relevant for its protective effect against OS, we performed a genetic epistasis analysis using mutant *C. elegans* strains. We first evaluated survival under lethal juglone-induced OS in strains lacking functional DAF-16/FOXO or SKN-1/Nrf2. *skn-1* mutants showed a ~ 57% reduction in locomotor activity—used here as a proxy for viability—compared to wild-type animals, as quantified by the area under the activity curve (AUC: 487 ± 187 AU for *skn-1* mutants vs. 1123 ± 349 AU for wild-type animals; Additional file 1: Fig. S2). This marked decline confirms their heightened sensitivity to OS. In contrast, *daf-16* mutants displayed survival profiles comparable to wild-type animals (AUC of 1018 ± 278 AU). Interestingly, hypomorphic *hsf-1* mutants were unexpectedly resistant to juglone (AUC of 1657 ± 351 AU, Additional file 1: Fig. S2), consistent with previous reports of age-dependent resistance in this strain [37]. Given that geraniol treatment actually downregulated the HSF‑1 target *hsp‑70*, and considering the complex, potentially confounding resistance of the *hsf‑1* mutant itself, we excluded this strain from further mechanistic analysis. This decision focuses our investigation on the pathways that geraniol robustly activates.

**Fig. 4:**
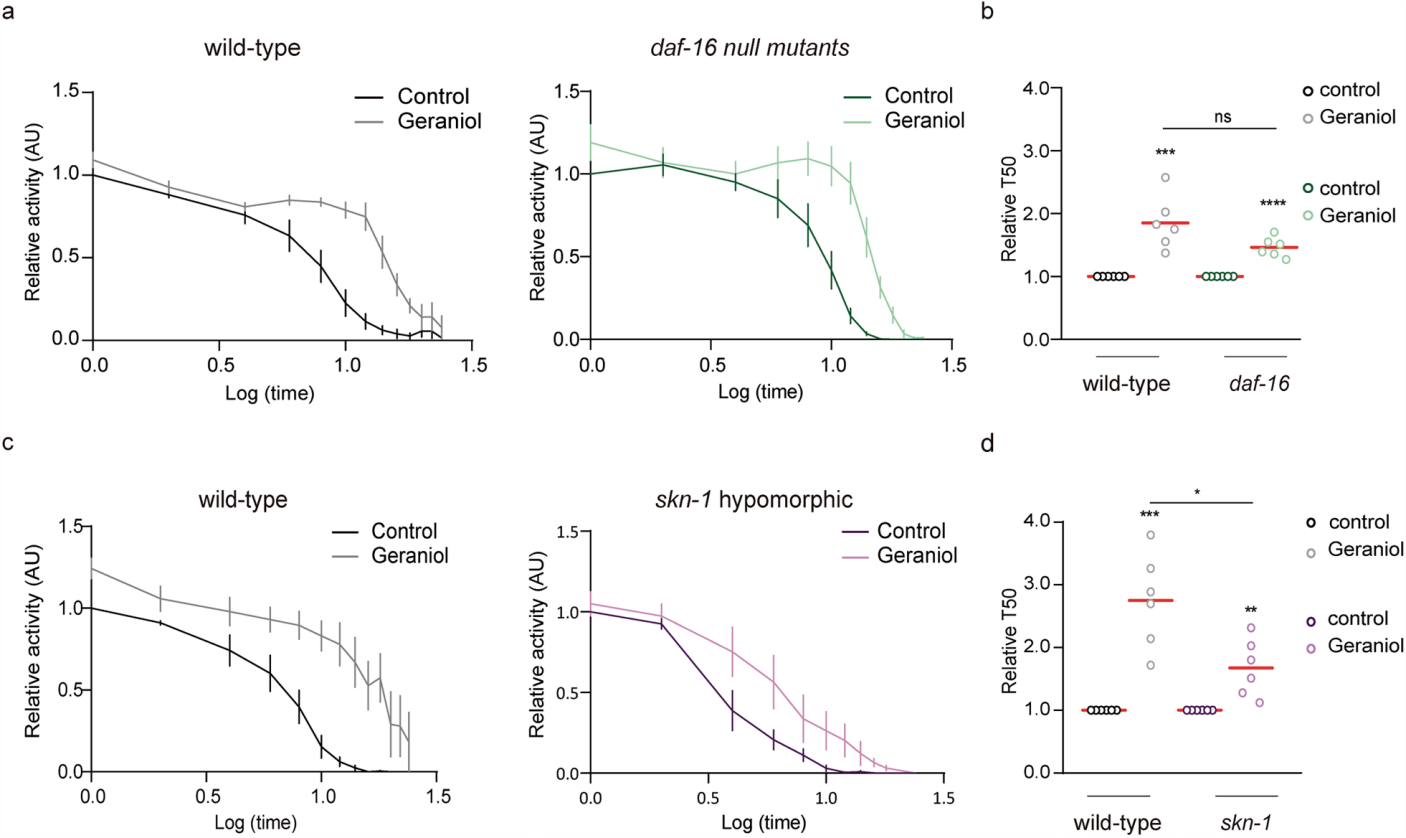
Geraniol protection against OS involves SKN-1. **a**–**d** Animal locomotion at the adult day 1 stage in the presence of the oxidizing agent juglone (240 μM). Animals were exposed throughout development to 1 mM geraniol (light colors) or vehicle (DMSO, dark colors). Results are presented as mean locomotor activity (AU) normalized to hour 1 of the control condition for each strain. **a** Wild-type (left, black line) and *daf-16* null (right, green line) animals. **b** Relative T50 comparison for both wild-type and *daf-16* mutants. **c** Wild-type (left, black line) and *skn-1 hypomorphic* (right, purple line) animals. **d** Relative T50 comparison for both wild-type and *skn-1* mutants. For **a**-**d**.The experiment was performed in 6 independent replicates, each including 8–12 wells (approximately 50 worms per well). Statistical significance was determined using Student's t-test (ns *p* > 0.05, *p < 0.05, **p < 0.01, ***p < 0.001, ****p < 0.0001)

We next asked whether geraniol could still protect these mutants from juglone-induced OS. Animals were pretreated with 1 mM geraniol or vehicle (DMSO) and then exposed to juglone. Geraniol significantly improved survival in *daf-16* mutants to an extent comparable to that in wild-type animals (Fig. 4a and b), indicating that DAF-16 is not essential for geraniol-mediated protection. In contrast, the protective effect of geraniol was significantly reduced in *skn-1* mutants (Fig. 4c and d), which remained highly sensitive to juglone.

Together, these results demonstrate that SKN-1/Nrf2 is key for geraniol’s antioxidant protection against OS, while DAF-16/FOXO—although activated by geraniol—is dispensable in this context.

### In silico pharmacokinetic profiling predicts favorable drug-like properties and bioavailability, supporting translational potential

Our genetic and phenotypic results in *C. elegans* establish geraniol as a potent activator of the conserved SKN-1/Nrf2 pathway, conferring significant resistance to OS. While invertebrate models provide powerful insights into molecular mechanisms, translation to therapeutic potential in mammals requires evaluation of a compound's pharmacokinetic profile—encompassing absorption, distribution, metabolism, and excretion (ADME). These properties determine whether a bioactive molecule can reach target tissues at effective concentrations in vivo and are critical predictors of clinical success. To bridge this translational gap and assess geraniol's viability as a lead compound, we performed comprehensive in silico ADME profiling using the SwissADME platform, a validated tool for predicting drug-likeness and bioavailability.

**Fig. 5:**
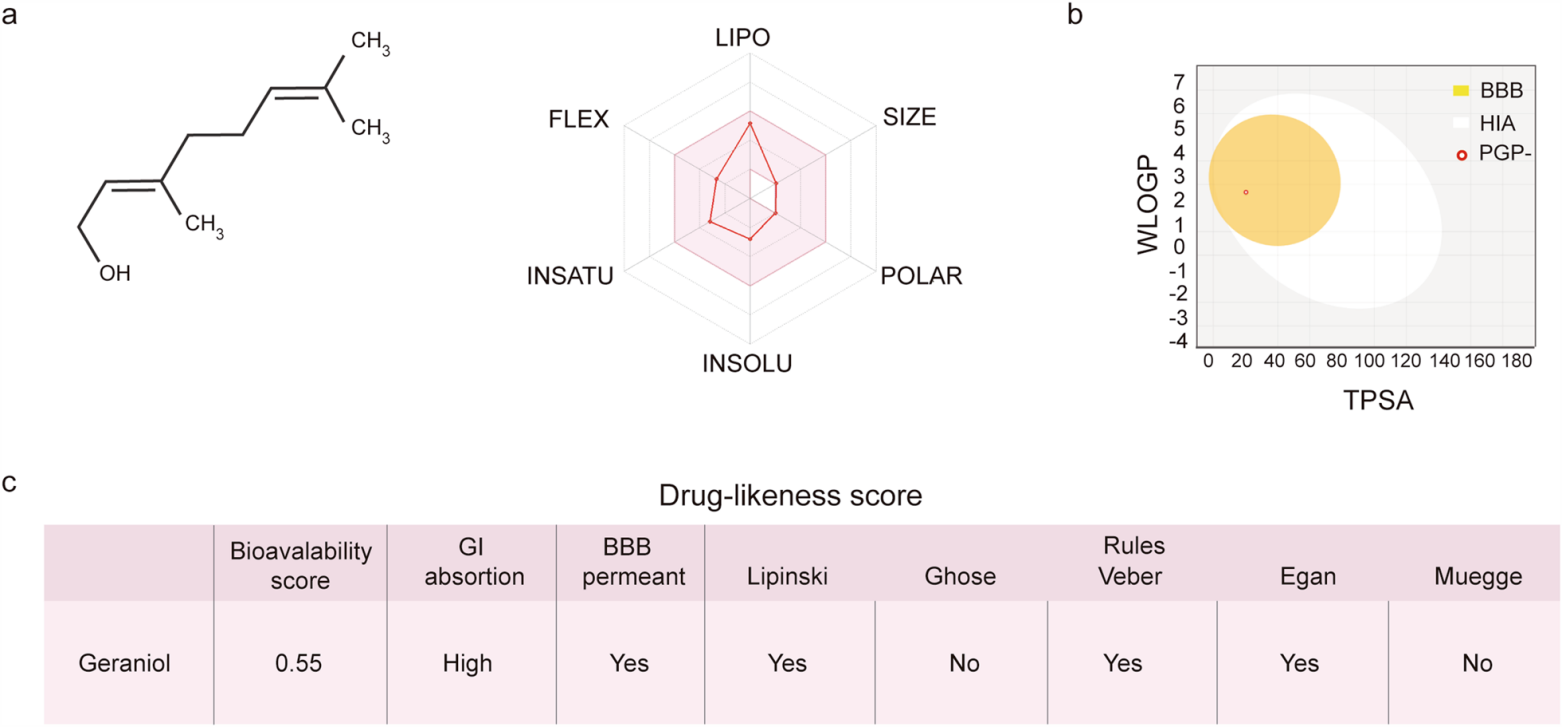
Predicted drug-likeness profile of geraniol using SwissADME. **a** Left: Chemical structure of geraniol. Right: Oral viability radar summarizes six key physicochemical properties—lipophilicity, molecular size, polarity, solubility, saturation, and flexibility—that collectively predict the likelihood of oral bioavailability. Geraniol falls within the optimal range for all these parameters (colored in pink), indicating favorable oral bioavailability. **b** BOILED-Egg (Brain Or Intestinal Estimated permeation predictive model) for geraniol. Geraniol (red point) falls within the yellow (Blood–Brain-Barrier: BBB) region, indicating a high probability of passive BBB permeation and human intestinal absorption (HIA). Geraniol is also predicted to be P-glycoprotein–negative (PGP −), suggesting it is not subject to efflux transport. WLOGP (y-axis) represents lipophilicity, and TPSA (x-axis) represents topological polar surface area. **c** Drug-likeness parameters of geraniol. Predictions were obtained after introducing SMILES structures in the SwissADME web server. Bioavailability score, Gastrointestinal (GI) adsorption and the Lipinski’s, Ghose’s, Veber’s, Egan’s and Muegge’s rules define the drug-likeness score for each compound

The software analysis showed that geraniol has a bioavailability score greater than zero, indicating that its physicochemical and pharmacokinetic profile is comparable to those of orally administered drugs with established clinical efficacy. The analysis predicted high gastrointestinal absorption, suggesting efficient uptake after oral administration. Importantly, the model also predicted significant blood–brain barrier permeability (Fig. 5b and c), highlighting geraniol's potential to exert protective effects within the central nervous system—a relevant feature for neurodegenerative conditions associated with OS.

Furthermore, examination of the oral bioavailability radar—which evaluates six fundamental physicochemical properties—revealed that geraniol's profile for lipophilicity, molecular size, polarity, solubility, flexibility, and saturation falls entirely within the optimal range defined for drug-like compounds (Fig. 5). These computational predictions collectively suggest that geraniol possesses favorable ADME characteristics, including metabolic stability and efficient membrane permeation. This analysis supports the notion that geraniol is not only bioactive in a model organism but also embodies key molecular properties necessary for further pharmacological development. Thus, our findings provide a strong rationale for advancing geraniol into preclinical mammalian studies aimed at mitigating OS-related pathologies.

## Discussion

Although plant extracts with antioxidant activity have shown promise in preclinical models, their clinical translation has often been disappointing. This is partly due to the inherent complexity of such extracts, whose composition varies with extraction methods, environmental factors, and plant physiology. Therefore, studying purified constituents is crucial to identify the active molecules, define their mechanisms of action, and establish the pharmacological basis needed for clinical development.

In this work, we focused on geraniol, a terpene found in more than 250 essential oils [10, 38]. Geraniol is widely recognized for its diverse biological properties, including antimicrobial [39, 40], insect-repellent [41], and anthelmintic activities [42]. Additional reports indicate that geraniol displays a wide array of biological activities [43], including in vitro antioxidant effects. Its favorable pharmacokinetic properties—such as high membrane permeability and low toxicity—further enhance its therapeutic potential. Using *Caenorhabditis elegans* as a model, we demonstrate that geraniol functions as a potent in vivo antioxidant, reducing basal OS and conferring robust protection against exogenous oxidative insults, primarily through activation of the conserved SKN‑1/Nrf2 pathway.

Redox imbalance is a hallmark of aging and numerous diseases [2, 3]; thus, understanding how geraniol modulates OS may reveal actionable therapeutic targets. We took advantage of the experimental strengths of *C. elegans*—a system with well-conserved stress-response pathways and genetically tractable networks—to dissect the molecular mechanisms underlying geraniol’s effects. Consistent with an antioxidant role, geraniol treatment significantly lowered basal reactive oxygen species (ROS) levels, indicating an improved redox homeostasis. While ROS play important signaling and homeostatic roles [44], their levels must be tightly regulated, as excess ROS can damage DNA, proteins, and lipids. The lower ROS accumulation in geraniol-treated animals suggests that geraniol enhances endogenous stress-management pathways [45]. Following acute OS induced by lethal juglone exposure, geraniol markedly increased survival and reduced ROS accumulation. These findings demonstrate that geraniol improves the organism’s capacity to manage redox balance, and consequently, to protect against oxidative damage, in both physiological and OS conditions [1–3].

To elucidate the protective mechanism, we first considered whether dietary restriction (DR) could be contributing to the observed effects. DR is a well-established modulator of lifespan and stress resilience [17, 30], and in *C. elegans* it is typically associated with reduced fat storage, as seen in canonical DR models such as *eat-2* mutants [46, 47]. However, geraniol treatment did not alter fat stores, indicating that its protective effects are unlikely to arise from DR-related metabolic adaptations.

We then investigated key cytoprotective transcription factors downstream of the insulin/insulin-like signaling (IIS) pathway [35, 36]. Using transgenic reporter strains, we found that geraniol robustly activates SKN-1 (the *C. elegans* homolog of mammalian Nrf2) and, to a lesser extent, DAF-16 (the homolog of FOXO). Specifically, geraniol strongly induced the expression of *gst-4*, a canonical SKN-1 target, and moderately increased *sod-3*, a DAF-16 effector. The activation of these OS–related networks provides a mechanistic basis for geraniol's protective effects. SKN-1/Nrf2 and DAF-16/FOXO regulate genes involved in antioxidant defense, glutathione metabolism, and proteostasis [48–50]. Although these factors can act synergistically, their interaction under OS is complex and context-dependent [51]. For example, FOXO can downregulate Nrf2 activity by reducing ROS and increasing expression of its inhibitor Keap-1 [52].

To determine which pathway is functionally required for geraniol-mediated protection, we performed genetic epistasis analysis. While geraniol improved survival in *daf-16* mutants to wild-type levels, its protective effect was significantly reduced in *skn-1* mutants. This indicates that SKN-1, but not DAF-16, is relevant for geraniol's antioxidant action against juglone-induced OS.

SKN-1 is essential for embryogenesis and, post-embryonically, activates Phase II detoxification genes in both constitutive and stress-dependent manners [53]. These enzymes support glutathione synthesis, ROS scavenging, and detoxification of xenobiotic metabolites [54, 55]. Similarly, mammalian Nrf2 regulates a core antioxidant and chemoprotective response across tissues [55, 56]. Consistent with this conserved role, *skn-1* mutants were more sensitive to juglone than wild-type animals (Additional file 1: Fig. S2). Analogously, Nrf2-deficient mice show heightened susceptibility to chemical toxicity and carcinogenesis and fail to respond to chemoprotective agents [57–60].

A secondary yet intriguing finding was the moderate but significant downregulation of *hsp-70*, a primary target gene of the cytoprotective HSF-1 pathway. This observation gains relevance in light of the unexpected OS resistance displayed by hypomorphic *hsf-1* mutants. While HSF-1 activation is typically considered protective, its partial inhibition appears to trigger a compensatory adaptation that enhances resilience [21]. This aligns with the concept that the inhibition of one pathway can increase the activity of another with similar or partially redundant functions [61, 62]. As HSP-70 functions as a molecular chaperone, reduced expression of this protein may increase the availability of unbound client proteins, including transcription factors, thereby facilitating their nuclear translocation and subsequent activation of gene expression [63]. Consequently, this phenomenon suggests that geraniol’s mechanism may involve a pharmacological rewiring of the cellular stress-defense network, whereby a mild repression of one axis (HSF-1) could potentiate the activation of another (i.e., SKN-1/Nrf2), resulting in a net protective outcome. This model, while consistent with our data, remains speculative and warrants further investigation to establish any potential causal relationship between HSF-1 modulation and the enhanced SKN-1 activity observed.

In conclusion, our study demonstrates that geraniol provides broad-spectrum antioxidant protection by enhancing basal redox homeostasis and specifically engaging the SKN‑1/Nrf2 detoxification axis upon challenge. Its ability to differentially regulate multiple conserved pathways—with SKN-1/Nrf2 serving as a pivotal and non-substitutable mediator—suggests potential for synergistic effects in complex disorders driven by OS. Given the demonstrated efficacy of antioxidant strategies in preclinical models, our findings support the translational potential of plant-derived compounds such as geraniol for mitigating OS–related pathologies.

## Materials and methods

### Reagents

Geraniol (98%) was purchased from Sigma-Aldrich (St. Louis, MO, USA) and DMSO (≥ 99%) was purchased from Biopack (CABA, ARG).

### *C. elegans* culture and maintenance

Worms were cultured and maintained on Nematode Growth Medium (NGM) agar plates seeded with *Escherichia coli OP50* as a food source [64, 65]. Worm culture and all the experiments were carried out at 20 °C, unless otherwise noted. The wild-type strain was Bristol N2. The strains were provided by the *Caenorhabditis Genetics Center* (CGC), which is funded by the NIH Office of Research Infrastructure Programs (P40 OD010440). Low population density was maintained throughout development and during the assays.

For all experiments, animals were age-synchronized. The strains used were: N2 (wild-type), JV1 *jrIs1 [rpl-17p::HyPer*+ *unc-119(*+*)],* CL2166 *dvIs19 [(pAF15)gst-4p::GFP::NLS] III,* BC10060 *dpy-5(e907) I; sEx884 [rCesC12C8.1::GFP*+ *pCeh361],* CF1553 *muIs84 [(pAD76) sod-3p::GFP*+ *rol-6],* TJ356 *zIs356 [daf-16p::daf-16a/b::GFP*+ *rol-6(su1006)],* QV225 *skn-1(zj15) IV,* PS3551 *hsf-1(sy441) I,* GR1307 *daf-16(mgDf50) I.*

### Geraniol exposure

The pharmacological assays were performed as described before [35, 66]. Geraniol was diluted in DMSO. As DMSO has an influence on relevant parameters, we excluded DMSO effects for our read-outs by adjusting the solvent concentration in all experimental groups to the respective concentration of the highest treatment group. In all experiments, DMSO did not exceed 0.2%, a concentration at which no self-effect was reported [67]. 1 mM geraniol stocks were prepared with DMSO and diluted into NGM agar. Plates were seeded with OP50 (with geraniol), stored at 4 °C and used within 1 week. In all experiments, age-synchronized individuals were used. To achieve this synchronization, gravid adults were placed on NGM plates containing geraniol, allowed to lay eggs for 3 h, and then removed. Descendants were maintained on these plates until the stage of interest.

### Locomotor activity

Adult day 1 worms (AD1) were transferred to 96 multiwell plates (50 worms per well) with M9 buffer and *E. coli OP50* culture at OD600 ≈ 0,1. Locomotor activity was then monitored as a proxy for viability using an infrared tracking system (WMicrotracker MINI, PhylumTech, Argentina), which detects infrared microbeam interruptions due to worm movement in liquid media [27].

### Oxidative resistance assay

Oxidative resistance was evaluated following the locomotion protocol described above, with one modification. After exposure to the drug, worms were transferred to 96 multiwell plates (50 worms per well) containing 240 µM juglone, a naphthoquinone derived from *Juglans regia* used as an oxidative stressor. Worm motility was assessed using the same infrared tracking device (WMicrotracker MINI, PhylumTech, Argentina) as a proxy for survival. N2 (wild-type), QV225 (*skn-1* hypomorphic mutant), PS3551 (*hsf-1* hypomorphic mutant), and GR1307 (*daf-16* null mutant) strains were evaluated under oxidative conditions to determine their importance for geraniol protection.

### Microscopy and image analysis

For microscopy analysis, animals were grown on NGM plates containing the compound. At the proper age for each experiment, worms were immobilized in sodium azide (0.25 M) and mounted onto slides with 2% agarose pads. Images were obtained using an epifluorescence microscope (NIKON ECLIPSE TE2000-S) coupled to a monochrome CMOS Nikon DS-Qi2 Camera (NIS-Elements acquisition software) at × 10, × 20 and × 40 mag. Images were analyzed using Image J FIJI software (ImageJ, National Institutes of Health, Bethesda, MD, United States).

### ROS production

The JV1 strain genetically expresses HyPer, a hydrogen peroxide–specific sensor composed of a circularly permuted yellow fluorescent protein (cpYFP) inserted into the regulatory domain of the prokaryotic H_2_O_2_-sensing protein OxyR. Upon selective and sensitive oxidation by H_2_O_2_, HyPer forms a disulfide bridge between the separated regions of the OxyR regulatory domain, thereby altering its fluorescent properties. Animals were age-synchronized and maintained on NGM plates containing either 1 mM geraniol or vehicle. Images were acquired at the L4 stage using excitation filters of 488 nm (oxidized) and 400 nm (reduced) [68]. For each animal, the Ox/Red ratio was calculated as a proxy for the redox state [28].

### Nutritional state

To assess fat storage in *C. elegans*, we followed the Oil-Red O (ORO) staining protocol described by Wang *et., al* [47]. Briefly, synchronized AD1 animals were fixed in paraformaldehyde, permeabilized by repeated freeze–thaw cycles, and incubated with freshly prepared ORO working solution. After incubation, worms were washed thoroughly to remove excess dye, mounted, and contrast-mode images were acquired for analysis. Quantification was performed in ImageJ FIJI software. For each animal, we measured the average pixel intensity within a 40-pixel-radius region immediately posterior to the pharynx. A 40-pixel-radius background region was also measured, and this value was subtracted from the staining intensity to obtain background-corrected measurements.

### *gst-4*, *hsp-70* and *sod-3* expression

*Gst-4* expression was analyzed in the transgenic strain containing a transcriptional GFP reporter: CL2166 *dvIs19 [(pAF15)gst-4p::GFP::NLS]* III; *hsp-70* expression was analyzed in the transgenic strain BC10060 *dpy-5(e907) I; sEx884 [rCesC12C8.1::GFP*+ *pCeh361]* and *sod-3* expression was analyzed in the transgenic strain CF1553 *muIs84 [(pAD76) sod-3p::GFP*+ *rol-6].*

Images of AD1 animals were taken at 10 × objective magnitude and mean fluorescence intensity was measured in the whole body (for GST-4 and HSP-70) or at 20 × for SOD-3 (head animals, same-sized Regions of Interest (ROIs)). Fluorescence intensity was quantified using ImageJ FIJI software and was relativized to the control condition for analysis.

### Subcellular DAF-16 localization

DAF-16 translocation was analyzed using strains containing the translational *Pdaf-16::DAF-16a/b::GFP* reporter. AD1 animals exposed to mild stress (200 µM juglone for 10 min) were analyzed. The number of GFP-labeled nuclei per animal was quantified using Image J FIJI software and normalized to the control condition within the day.

### In silico evaluation of ADME profile

Geraniol structure and SMILES (Simplified Molecular Input Line Entry System) notation were generated using the structure file generator available in the Open Chemistry Database of the National Institutes of Health (NIH) (https://pubchem.ncbi.nlm.nih.gov/). The resulting SMILES string was exported to the free online tool SwissADME (http://www.swissadme.ch/) [24, 69]. Considering six physicochemical properties such as lipophilicity, molecular size, polarity, solubility, saturation and flexibility, this tool predicts and weights the probability of the oral bioavailability of the drug by building a bioavailability radar. BOILED-Egg graph was also generated to evaluate the capacity of geraniol to passively permeate through the blood–brain barrier.

### Statistical analysis

The results presented in each figure are the average of at least three independent assays. Bars represent mean ± SEM. All the statistical tests were performed after checking normality. Grubbs’ test was used for outlier analysis (*p* < 0.05). Typically, one-way analysis of variance was employed for analyzing multiple parametric samples, and Holm–Sidak’s post hoc test was used for comparisons against a control group. In cases where comparisons were made between two independent conditions, a Student's *t*-test was utilized for parametric data, while the Mann–Whitney *U* test was employed for non-parametric data. For T50 analysis, we fitted using Prism 8.0.1 (GraphPad Software Inc., La Jolla, CA, USA) to a Hill equation with four parameters. The statistical information is indicated in the figure legends.

## Supplementary Information

Additional file 1.

## Data Availability

The data that support the findings of this study are available in the following link: https://osf.io/2fp6s
