# Supplementary material for: Natural monoterpenoid geraniol promotes antioxidant defense and stress tolerance via SKN-1/Nrf2 activation in Caenorhabditis elegans
Source: Nat Prod Bioprospect. 2026 Apr 22;16(1):56. doi: 10.1007/s13659-026-00604-4 (PMC13100220; doi:10.1007/s13659-026-00604-4)
Supplement: Supplementary file 1 — Additional file 1. [file 13659_2026_604_MOESM1_ESM.pdf]

Supplementary Figures and legends

Supplementary Figure 1

A

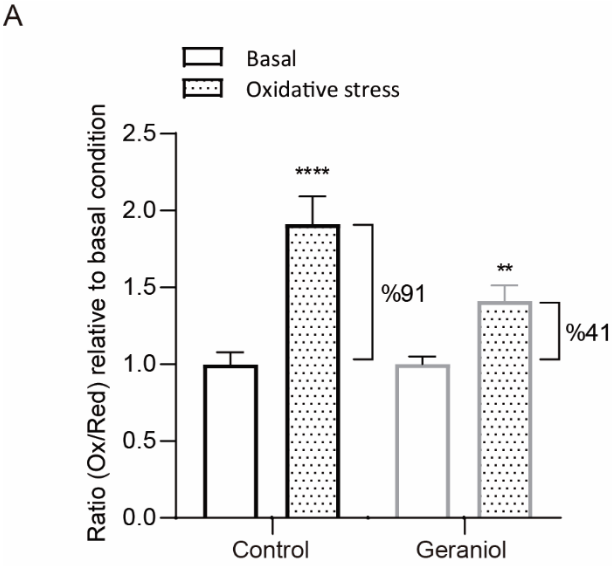

Bar graph showing intracellular ROS levels under basal and oxidative stress conditions. Animals were treated with vehicle (DMSO) or 1 mM geraniol. For each treatment, ROS levels were measured under basal conditions and after oxidative stress induction with juglone (200 µM, 20 min). Intracellular ROS levels are expressed as the oxidized/reduced HyPer ratio. Data are presented relative to the corresponding basal (non-stressed) condition for each treatment group

Supplementary Figure 2

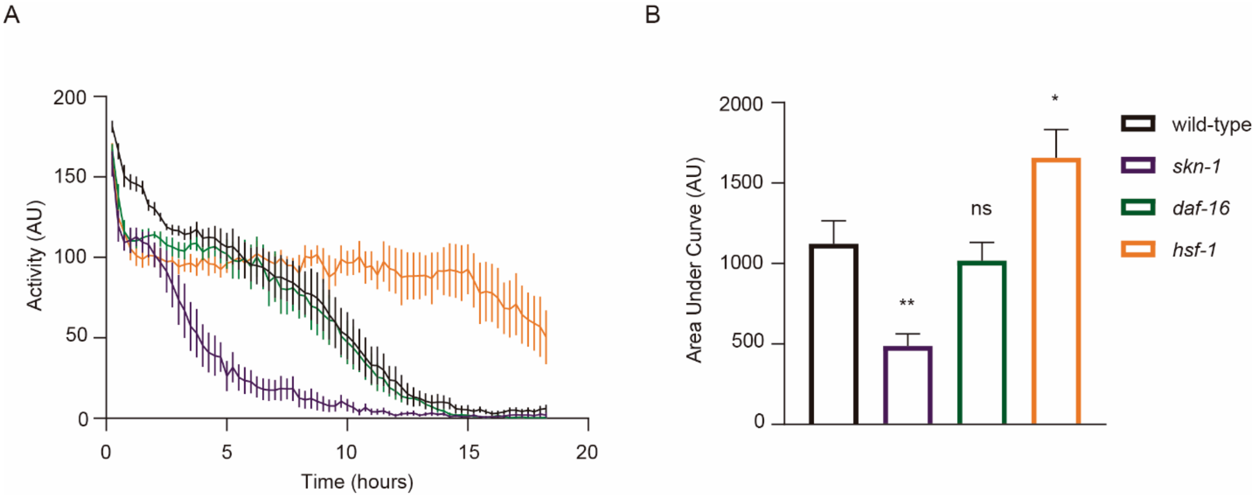

a) Locomotor activity (Arbitrary Units: AU) of wild-type (Black line), *daf-16* (green line), *skn-1 (purple line),* and *hsf-1* (orange line) mutant animals under oxidative stress (juglone 240 µM) at the adult day 1 stage, measured using the WMicroTracker. Experiments were performed in at least 3 independent replicates, each including 6–12 wells per strain (approximately 50 worms per well). **b)** Area under the curve (AUC) quantification (AU) for each strain. Statistical significance was assessed using one-way ANOVA with comparisons against wild-type animals. Ns: no significant differences, * ($p < 0.05$), ** ($p < 0.01$)
